# Mechanism Underlying Selective Albuminuria in Minimal Change Nephrotic Syndrome

**DOI:** 10.1155/2019/5859102

**Published:** 2019-11-03

**Authors:** Akihiro Tojo

**Affiliations:** Department of Nephrology & Hypertension, Dokkyo Medical University, Mibu, Tochigi, Japan

## Abstract

As water and solutes are filtered through the slit membrane, it is an a priori concept that a slit membrane is an essential filtration barrier for proteins, including albumin. However, in cases of minimal change nephrotic syndrome, the number of slit membranes is reduced by the foot process effacement and tight junction-like cell adhesion. Furthermore, albumin endocytosis is enhanced in the podocytes under condition of minimal change disease, and albumin is selectively transported by the albumin receptor FcRn. Suppressing the endocytosis of albumin with anti-FcRn antibody decreases the urinary protein level. The expression of motor molecules, such as cytoplasmic dynein 1 and myosin IX, is increased in the podocytes under conditions of minimal change nephrotic syndrome, suggesting the enhanced transport of vesicles containing albumin. Podocyte vesicle transport may play an important role in the pathology of selective albuminuria in cases of nephrotic syndrome.

## 1. Introduction

It is important to understand the mechanism underlying proteinuria and albuminuria because they are early detection markers of renal diseases. Proteinuria is classified into three types: glomerular proteinuria, tubular proteinuria, and overflow proteinuria with abnormally increased levels of plasma protein, such as Bence Jones protein or myoglobin [1, 2]. Tubular proteinuria caused by tubular dysfunction of protein reabsorption in Fanconi syndrome [3, 4], Dent's disease [5], or tubulointerstitial nephritis is characterized by the presence of low-molecular-weight proteins (LMWPs), including *β*2-microglobulin, light chain, *α*1-microglobulin, and retinol binding proteins [1, 6]. Microalbuminuria in early-stage diabetes is also associated with reduced albumin reabsorption via megalin in the proximal tubule [7, 8]. Tamm–Horsfall protein (THP, uromodulin) is a major protein in the urine excreted from the loop of Henle [9]. Glomerular protein is further divided into three types: nonselective proteinuria with hematuria, nonselective proteinuria without hematuria, and selective proteinuria.

The mechanism underlying selective proteinuria used to be ascribed to dysfunction of the slit membrane due to a reduction in nephrin or reduced negative charge at the glomerular basement membrane (GBM) in patients with minimal change nephrotic syndrome (MCNS) [10–12]. However, it is difficult to determine the morphological pathway of albumin filtration when the cell body or primary process of the podocyte covers the GBM with tight junction-like cell adhesion in cases of MCNS, even if the reduction in nephrin of the slit membrane and negative charge at the GBM play important roles in selective albuminuria.

This chapter will focus on the relationship between the morphological changes and functional mechanisms of proteinuria and discuss a new aspect of the mechanism underlying selective albuminuria in MCNS.

## 2. Electrophoresis of Urinary Proteins in Various Renal Diseases

**Figure 1:**
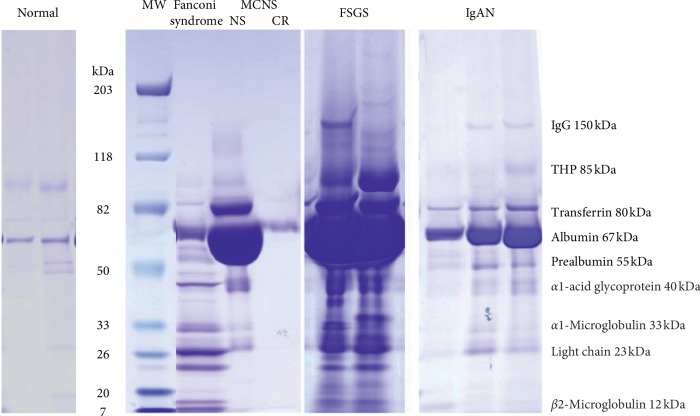
SDS-polyacrylamide gel electrophoresis in various renal diseases including Fanconi syndrome, minimal change nephrotic syndrome (MCNS), focal segmental glomerulosclerosis (FSGS), and IgA nephropathy (IgAN). MW: molecular weight marker; NS: nephrotic syndrome; CR: complete remission; THP: Tamm–Horsfall protein.

Proteins are filtrated through the glomerular capillary wall and secreted from tubules before finally being excreted into the urine. Sodium dodecyl sulfate-polyacryl amide gel electrophoresis (SDS-PAGE) was performed to separate urinary proteins according to their molecular weight in normal control samples and samples from patients with various renal diseases (Figure 1). In normal urine, the most abundant proteins are albumin (67 kDa) and THP (uromodulin, 85 kDs), and high-molecular-weight proteins (HMWPs) like IgG (150 kDa) and LMWPs including *α*1-microglobulin (33 kDa), light chain (23 kDa), and *β*2-microglobulin (12 kDa) are only faintly observed. Under normal conditions, LMWPs are filtrated through glomeruli with a high sieving coefficient of 0.987 and reabsorbed 82% in the proximal tubule and 10% in the distal nephron. Therefore, only a small amount of LMWPs excreted into the final urine [13, 14]. In Fanconi syndrome, the megalin-receptor-mediated endocytosis of LMWPs is disturbed, resulting in the increased excretion of albumin, prealbumin (transthyretin, 55 kDa), *α*1-acid glycoprotein (40 kDa), and other LMWPs (Figure 1). In contrast, nonselective proteinuria observed in focal segmental glomerulosclerosis (FSGS) or IgA nephropathy results in the increased excretion of HMWPs like IgG, as well as LMWPs (Figure 1). Selective proteinuria observed in MCNS resulted in the increased excretion of albumin, transferrin (80 kDa), and prealbumin (55 kDa), and interestingly, not only IgG but also LMWPs were ultimately not excreted into the urine (Figure 1). A clearance study with dextran of various sizes also showed a reduction in the fractional dextran clearance of smaller-size dextran (less than 50 Å in molecular radius) in MCNS compared with the normal control sample [11]. In normal glomerulus, the slit membrane is a continuous junctional band between foot processes with 350∼400 Å width, and there are rectangular pores approximately 40 by 140 Å in cross-section and 70 Å in length, that is, just a size of albumin molecule [15]. The slit pores are composed of nephrin molecules and their associated proteins, and mutations in the slit membrane-associated proteins are believed to cause proteinuria with enlarged slit pores [12, 16]. The phenomenon of decreased LMWP excretion in MCNS is difficult to attribute to an increase in the slit membrane pore size associated with nephrin mutations, or to a reduced negative charge in GBM resulting in the expulsion of negative charged proteins. The smaller LMWPs can pass through the enlarged slit pores more easily than albumin, despite having a negative charge like albumin. Thus, selective albuminuria cannot be attributed to a disorder of the slit membrane or a reduced negative charge at the GBM.

## 3. Morphological Changes in Podocytes Correlated with the Classification of Glomerular Proteinuria by SDS-PAGE

**Figure 2:**
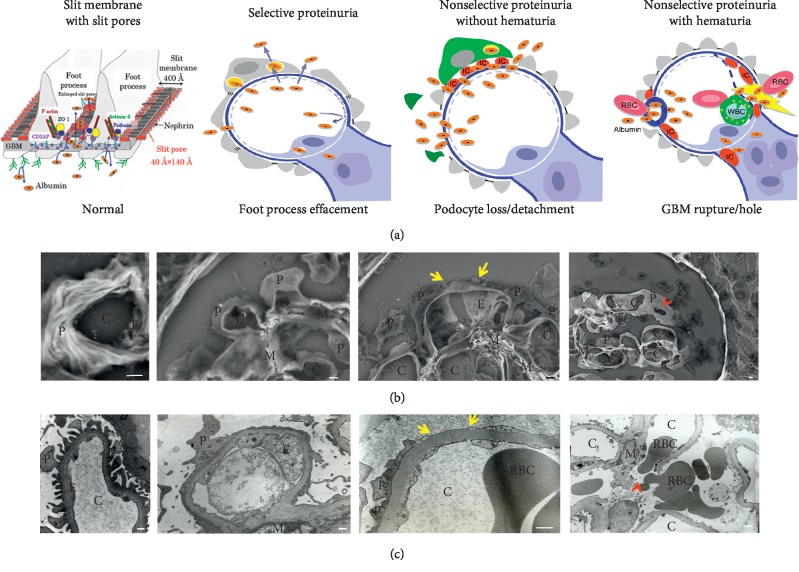
Morphological changes of the glomerular filtration barrier and selectivity of proteinuria. (a) A schematic illustration. (b) A micrograph of a glomerulus in a paraffin section obtained using a low-vacuum scanning electron microscope (LVSEM) in each group of proteinuria selectivity. (c) A micrograph of the glomerular capillary wall obtained using a transmission electron microscope (TEM) in each group of proteinuria selectivity. Selective albuminuria is usually found in patients with minimal change nephrotic syndrome, nonselective proteinuria without hematuria associated with podocyte detachment (yellow arrows) is found in patients with membranous nephropathy, focal segmental glomerulosclerosis, and diabetic nephropathy, and nonselective proteinuria with hematuria associated with GBM rupture/hole (red arrowheads) is found in patients with IgA nephropathy, ANCA-related glomerulonephritis, poststreptococcal acute glomerulonephritis, and membranoproliferative glomerulonephritis. P: podocyte; E: glomerular endothelium; C: glomerular capillary; M: mesangium; RBC: red blood cell. Scale bars indicate 2 *μ*m on the LVSEM micrograph and 500 nm on the TEM micrograph.

Morphological changes in the capillary wall are correlated with the proteinuria profiles observed by SDS-PAGE. Transmission electron microscopy (TEM) showed narrow foot processes with slit membrane between the foot processes under normal proteinuria. The low-vacuum scanning electron microscopy (LVSEM) observation of light microscopic paraffin-embedded sections showed three-dimensional features of foot processes with a narrow width that covered the capillary wall (Figure 2). In one case of nonselective proteinuria without hematuria, the podocyte was found to be detached from the GBM on TEM observation; however, it is very difficult to detect these lesions because only a limited area of glomerulus can be observed by TEM. On the other hand, podocyte loss or detachment was easily detected using an LVSEM, which enabled the observation of all glomeruli in whole paraffin-embedded sections with periodic acid-methenamine-silver (PAM) staining (Figure 2). Podocyte loss or detachment resulted in the increased excretion of HMWPs as well as albumin and LMWPs in FSGS, membranous nephropathy, and diabetic nephropathy [11, 14, 17].

In one case of nonselective proteinuria associated with hematuria, ruptures or holes in the GBM were noted on TEM observation [17]. The disruption of the GBM is necessary for red blood cells to pass through; however, it is difficult and time-consuming to detect GBM rupture by TEM because the observation area is limited to part of a glomerulus. LVSEM is therefore more suitable for detecting holes in the GBM in cases of IgA nephropathy, ANCA-related glomerulonephritis, or membranoproliferative glomerulonephritis (Figure 2). All plasma proteins leaked through the holes in the GBM and the bands of proteins of HMWPs, albumin, and LMWPs were observed in the urine of a patient with nonselective proteinuria with hematuria. In contrast, it is difficult to find the filtration pathway of proteins when a patient with MCNS shows selective albuminuria because TEM shows that the podocyte cell body and primary process with foot process effacement cover the GBM wall in MCNS, the slit pore density is reduced by a maximum of 80%, and half of the slit membrane between the foot processes becomes a tight junction-like structure [18]. Thus, we suggested that massive albumin might be transported through podocyte cell bodies in cases of MCNS.

## 4. Percentage of Foot Process Effacement and Acute Kidney Injury (AKI) in Minimal Change Nephrotic Syndrome

**Figure 3:**
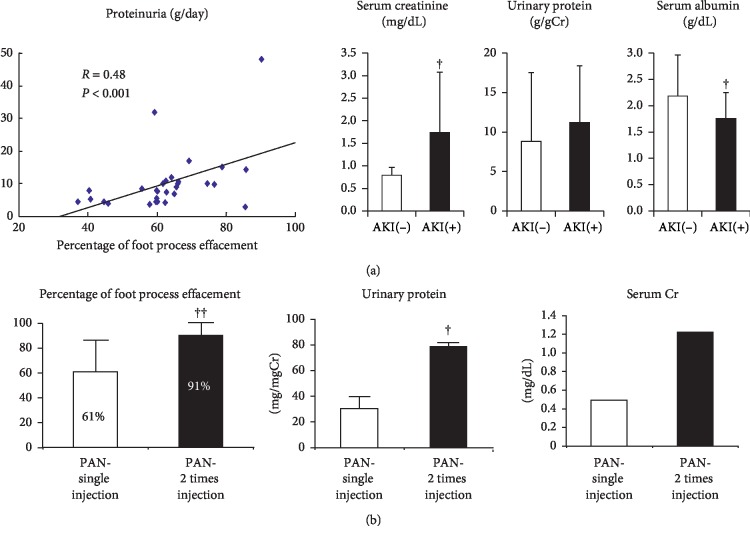
Correlation between the percentage of the area along the capillary wall with foot process effacement and proteinuria in human patients with minimal change nephrotic syndrome with or without acute kidney injury (AKI) (a) and an animal model of minimal change nephrotic syndrome induced by a single or two injections of puromycin aminonucleoside (PAN, *n* = 5 in each) (b). Patients with MCNS with AKI (*n* = 13) showed significantly higher levels of serum creatinine and lower levels of serum albumin at biopsy than MCNS patients without AKI (*n* = 25).

**Figure 4:**
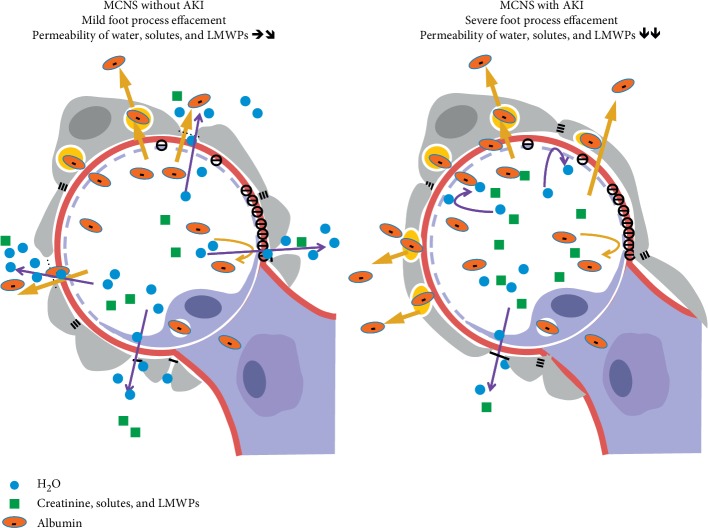
A schematic illustration of glomerular podocyte effacement in minimal change nephrotic syndrome with or without acute kidney injury.

The correlation of the severity of process effacement with the degree of proteinuria is controversial [19–21]. Our data from 38 patients with MCNS indicated a positive correlation between the percentage of foot process effacement and the degree of proteinuria (Figure 3). Of these patients, 13 (34%) developed AKI and showed more severe proteinuria and lower serum albumin and serum total protein levels than the 25 MCNS patients without AKI (Figure 3). This finding may suggest that podocytes with foot process effacement enhanced the albumin transport through the podocyte cell body but decreased the water and creatinine filtration through the tight junction-like slit membrane in MCNS with AKI. Clinically, the renal hypoperfusion due to severe hypoalbuminemia in MCNS is considered as a major cause of AKI. To elucidate the relationship between foot process effacement and proteinuria or AKI, we studied an animal model of MCNS induced by puromycin aminonucleoside (PAN) injection. Rats that received two injections of PAN at weekly intervals clearly showed increased serum creatinine levels associated with increased foot process effacement and proteinuria compared to rats that received a single injection of PAN (Figure 3). These data suggest that the slit membrane functions as a filtration barrier for water and small molecules, including electrolytes, creatinine, and LMWPs; thus, severe foot process effacement with reduced slit membrane may decrease this clearance and may cause AKI, whereas albuminuria increased via enhanced podocyte albumin transcytosis in rats with twice injection of PAN. Further studies are necessary to visualize a water and creatinine filtration through slit membrane. This hypothesis is presented as a schematic figure (Figure 4), which may also explain why the levels of LMWPs were not increased in MCNS, as shown in Figure 1.

## 5. Evidence of Podocyte Albumin Transcytosis

For the past 50 years, protein endocytosis has been observed in the podocyte of nephrotic syndrome using electron microscopy [22–25]. However, most previous studies on this subject have used ferritin, horseradish peroxidase, or dextran as a tracer, so the albumin transport by podocyte has not been well investigated. To clarify the transcytosis of albumin, we attempted to visualize the albumin molecules by labeling them with 8 nm gold particles; however, the labeled albumin particles were too big to pass through the GBM [26]. We therefore used fluorescein isothiocyanate (FITC) labeled albumin (MW: 389 Da), which was observed in the podocytes of PAN nephrotic rats. However, FITC labeling easily dissociates from albumin, so some free FITC may have passed through the GBM [26]. We finally decided to use Evans blue (EB) labeled albumin (MW: 961 Da), as this agent strongly binds to albumin, with <0.001% dissociated free EB when the binding rate was 3 molecules of EB to 1 molecule of albumin [27]. When EB-albumin with red fluorescence was injected into green fluorescence protein (GFP) transgenic rats, the podocytes stayed in the green GFP fluorescence emission in control GFP rat. In contrast, the podocytes turned yellow after EB-albumin injection in GFP rats with MCNS induced by PAN [27]. Furthermore, there was an initial delay of approximately 3 to 5 minutes before EB-albumin appeared in the proximal tubules, suggesting that EB-albumin was filtrated at the glomerular capillary by transcytosis through the podocyte cell body [27]. Immunoelectron microscopic observation has confirmed the presence of EB-labeled albumin in the vesicles of podocytes in MCNS [27]. Podocytes have a large capacity for albumin endocytosis, with a *V*_max_ of 97.4 *μ*g/mg cell protein/h [28], and the total endocytosis capacity in human kidneys has been calculated to be 3.6 g/day, which is consistent with the daily glomerular filtration of albumin estimated in a micropuncture study [17, 29].

Several potential mechanisms underlying podocyte albumin endocytosis and transcytosis have been proposed, including clathrin-mediated endocytosis [30], caveolin-mediated endocytosis [31], fluid-phase endocytosis [32], and FcRn-mediated transcytosis [33]. Pitstop 2, an inhibitor of clathrin-mediated endocytosis, did not block albumin endocytosis in human cultured podocytes, whereas nystatin, an inhibitor of caveolin-mediated endocytosis, suppressed albumin endocytosis [31]. In an *in vivo* study, we showed that the administration of antibody for FcRn reduced albuminuria by approximately 50% in MCNS [27]. It was recently shown that fluid-phase endocytosis of free fatty acid-bound albumin takes place in podocytes [32]. These findings suggest that FcRn-dependent transcytosis, caveolin-dependent endocytosis, and fluid-phase macropinocytosis may play an important role in podocyte albumin endocytosis and transcytosis. In the renal transplantation study using podocyte-targeted FcRn knockout mice and wild type mice, Sarav et al. reported that podocyte FcRn reclaims albumin from urinary space and maintains serum albumin levels [34]. Further studies are necessary to elucidate the possibility of bidirectional transport of albumin by podocytes.

## 6. Podocyte Vesicle Transport by Dynein

**Figure 5:**
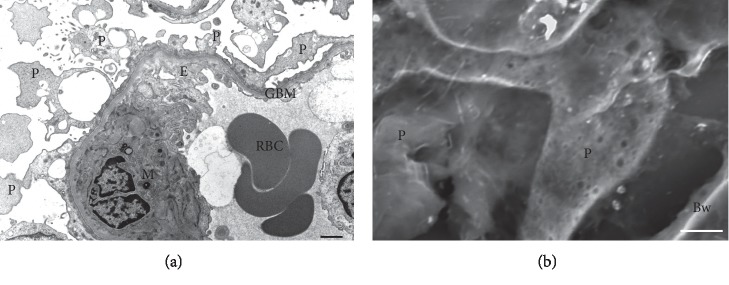
A micrograph of podocytes in a patient with minimal change nephrotic syndrome obtained using a transmission electron microscope (a). A micrograph of the podocyte surface in a patient with minimal change nephrotic syndrome obtained using a low-vacuum electron microscope with PAM staining section followed by 1% Ponceau S staining (b). P: podocyte; E: glomerular endothelium; C: glomerular capillary; M: mesangium; RBC: red blood cell; GBM: glomerular basement membrane; Bw: Bowman's capsule. Scale bars indicate 2 *μ*m.

**Figure 6:**
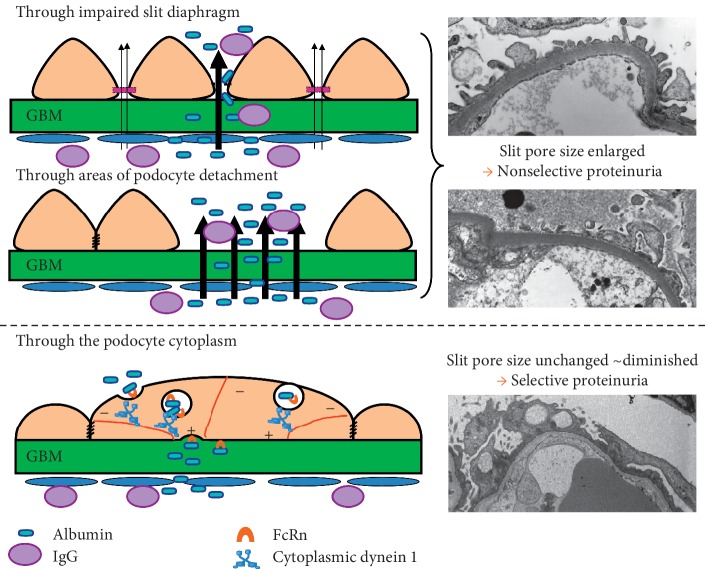
Putative mechanism of selective albuminuria in minimal change nephrotic syndrome.

Recent emerging evidence supports the notion of albumin transport through the podocyte cell body [26, 27, 30, 32, 35]. In 1955, Rinehart proposed that the glomerular filtrate is transported across the glomerular epithelial cytoplasm via small vesicles [36]. Large numbers of endocytic vesicles have been detected in the podocytes of MCNS patients by TEM [35, 37, 38]. Furthermore, a numerous holes have been observed at the basal and apical surface of podocytes [27, 39], suggesting endocytosis and exocytosis of transported proteins (Figure 5). Podocytes resemble neurons, and their primary process and foot process are similar to axons and dendrites. In neuronal axonal transport, kinesin-1 mediates the anterograde transport of synaptic vesicles, secretory vesicles, and mitochondria, whereas cytoplasmic dynein retrogradely transports cargo such as injured signaling endosomes, lysosomes, lipid droplets, and mitochondria from axonal lesion sites toward the soma along microtubules [40, 41]. Interestingly, an analysis of glomerular proteins by mass spectrometry revealed that levels of motor proteins including cytoplasmic dynein 1, myosin IXa (Myo9a), and myosin VIIb were increased in PAN-induced nephrotic rats compared with control rats [35]. As the minus end of the microtubule connects with the adherens junction, which is located below the tight junction of podocytes with foot process effacement, and cytoplasmic dynein 1 carries endocytosed vesicles toward the minus ends of microtubules, cytoplasmic dynein 1 will transport vesicles from the basal membrane to the apical membrane of podocytes (Figure 6). The mechanisms and morphological changes associated with podocytes in nonselective proteinuria and selective albuminuria are summarized in Figure 6. Further studies will be necessary to clarify the mechanism underlying selective albuminuria in MCNS.

## 7. Conclusion

The mechanism underlying selective albuminuria has not been sufficiently clarified; however, several pieces of evidence from animal models indicate that FcRn-dependent albumin transcytosis is increased in minimal change nephrotic syndrome. The numbers of endocytosed vesicles are increased under conditions of nephrotic syndrome, and these vesicles may be transported by motor proteins, including cytoplasmic dynein 1 and myosin IXa, whose expression is increased in glomeruli in cases of minimal change nephrotic syndrome.
